# Cash-like vouchers improve psychological well-being of vulnerable and displaced persons fleeing armed conflict

**DOI:** 10.1093/pnasnexus/pgac101

**Published:** 2022-06-30

**Authors:** John Quattrochi, Ghislain Bisimwa, Peter van der Windt, Maarten Voors

**Affiliations:** Department of Public Health, Simmons University, 300 The Fenway, Boston, MA 02115, USA; Department of Public Health, Catholic University of Bukavu, Bugabo, Avenue de la Mission 02, Kadutu Commune, Bukavu, Democratic Republic of Congo; Social Science Division, New York University Abu Dhabi, Saadiyat Island, PO Box 129188, Abu Dhabi, United Arab Emirates; Development Economics Group, Wageningen University and Research, Hollandseweg 1, 6706 KN, Wageningen, The Netherlands

**Keywords:** randomized control trial, humanitarian assistance, trauma, cash transfer, global health

## Abstract

The psychological burden of conflict-induced displacement is severe. Currently, there are 80 million displaced persons around the world, and their number is expected to increase in upcoming decades. Yet, few studies have systematically assessed the effectiveness of programs that assist displaced persons, especially in settings of extreme vulnerability. We focus on eastern Democratic Republic of Congo, where myriad local armed conflicts have driven cycles of displacement for over 20 years. We conducted a within-village randomized field experiment with 976 households, across 25 villages, as part of the United Nations’ Rapid Response to Population Movements program. The program provided humanitarian relief to over a million people each year, including vouchers for essential nonfood items, such as pots, pans, cloth, and mattresses. The vouchers led to large improvements in psychological well-being: a 0.32 standard deviation unit (SDU) improvement at 6 weeks, and a 0.18 SDU improvement at 1 year. There is no evidence that the program undermined social cohesion within the village, which alleviates worries related to programs that target some community members but not others. Finally, there was no improvement in child health.

Significance Statement Conflicts and disasters leave millions of people in urgent need of assistance. Humanitarian aid increasingly involves cash-based modalities. However, there is limited evidence on their impact because studies in these environments face considerable challenges related to logistics, fragility, security, and ethics. We show that a low-cost unconditional economic transfer program to individuals displaced by violence and vulnerable members of their host communities improves psychological well-being in the short and longer run. Similar transfer programs could be attractive to policymakers and humanitarian organizations working in contexts of extreme vulnerability. This study also demonstrates that randomized evaluations can be used to investigate the impact of interventions in humanitarian settings.

## Introduction

Humanitarian crises currently affect over 215 million people worldwide (1), and the psychological burden of conflict-induced humanitarian crises is severe (2). For example, the prevalence of mental disorders (depression, anxiety, post-traumatic stress disorder (PTSD), bipolar disorder, and schizophrenia) is over 20% in conflict settings, more than three times greater than nonconflict settings (3). A total of 1 in 10 people affected by conflict has a moderate to severe mental disorder at any point in time, and conflict creates a 5-fold increase in years lost to disability due to depression and PTSD (3).

Humanitarian interventions that provide direct economic assistance, such as cash transfers, have become popular among humanitarian actors over the past 15 years (4). In noncrisis settings, there is growing evidence that economic assistance improves psychological well-being (5–7). In humanitarian emergency settings, however, the evidence is scarce for a number of reasons: populations are often highly mobile; insecurity may prevent the research team from accessing study participants; and robust study designs may not be feasible for ethical or logistical reasons (8, 9). There are good reasons, however, to believe that economic assistance in humanitarian emergencies may have different effects than in noncrisis settings.

On the one hand, economic assistance has the potential to address some of the mechanisms through which conflict harms psychological well-being. Forcibly displaced households are often unable to transport many essential assets as they seek safety, which increases the daily stressors associated with basic survival. Displacement is associated with decreased household consumption (10, 11), reduced social cohesion (12), lower self-reported physical health, increased hypertension and tachycardia (13), and higher crude mortality rates (14). Economic assistance could increase resilience to these harms by allowing recipients to meet their most pressing needs for food, medicine, clothing, and everyday tasks like cooking and cleaning, which can in turn reduce daily stressors and protect dignity.

On the other hand, economic assistance may not address the causes of some psychological harms, such as trauma, violence, and fear. These factors may require specialized psychological care (15). Furthermore, economic assistance may reduce social cohesion if community members who do not receive assistance resent those who do, particularly in settings where tensions are already high due to conflict and the increased burden of hosting displaced individuals (16, 17).

To advance our understanding of the effectiveness of economic interventions to improve psychological well-being in humanitarian settings, we collaborated with the largest humanitarian assistance program in the Democratic Republic of Congo (DRC). Between 2004 and 2018, the program reached over 1 million people per year. To evaluate the program's economic assistance component, 976 internally displaced individuals (IDPs) and vulnerable households, across 25 villages, were randomly assigned to economic assistance or to a control group, which received no transfers. Those households assigned to treatment, received unconditional transfers of vouchers worth US$55 to US$90, which could be used to obtain essential household items (EHI) at a one-off fair organized by the program. We measured outcomes in the short (6 weeks) and longer (1 year) term, which are time scales relevant to humanitarian and development actors, respectively.

We find that economic assistance improved recipients’ psychological well-being after 6 weeks and after 1 year. We find no effects on child health, despite the potential for voucher purchases to improve water quality, facilitate food preparation, reduce exposure to mosquitoes, or be exchanged for medicine. We also find no evidence of a negative impact on social cohesion, suggesting that targeting some households but not others did not undermine local community relations. We find that the program increased asset ownership and dietary diversity, suggesting that household resilience may be a mechanism via which economic assistance improves psychological well-being. We also find, surprisingly, that economic assistance increased debt and the consumption of alcohol or tobacco.

Overall, our findings suggest that economic transfers can reduce the psychological harm caused by conflict and forced displacement. The intervention under study was low-cost, at about US$21 per beneficiary (about US${\$}$137 per household). Our results, thus indicate that economic assistance programs could be attractive to policy makers and humanitarian organizations working with vulnerable populations affected by violence and forced displacement.

## Research Design

We partnered with the Rapid Response to Movements of Population (RRMP) program, jointly managed by the United Nations Children's Fund (UNICEF) and the United Nations Office for the Coordination of Humanitarian Affairs (UNOCHA) in the DRC. RRMP provides humanitarian assistance, including vouchers for EHI. RRMP's core objectives are to improve well-being and reduce vulnerabilities of displaced people (including returnees) and vulnerable households in host communities. RRMP typically reaches over 1 million people per year. We carried out a randomized controlled trial during 2017 to 2018 in Congo's North Kivu province with a 1:1 allocation ratio to vouchers or a control group.

All participants provided verbal informed consent. We obtained ethical review approval from Catholic University of Bukavu (UCB/CIE/NC/006/2017) and Institutional Review Board (IRB) approval from New York University Abu Dhabi (#064–2017).

## Participants and Random Assignment

Across seven intervention sites, comprising 25 villages that recently began hosting displaced persons, RRMP staff conducted brief interviews with all households. Household vulnerability scores were calculated by RRMP based on ownership and quality of water containers, pans, buckets, farm tools, mattresses, sheets, and women's and children's clothing, as well as the number of household members with physical disabilities and children raised by a single parent. As a function of the amount of program resources available and the level of need for each site, RRMP set a vulnerability cut-off score. Those households with scores above the cut-off received assistance; others did not.

For this study, we recruited additional households with vulnerability scores immediately below the cutoff, i.e. households that would otherwise not have received assistance. Within each village, we then randomly assigned these study households to a treatment group (voucher) or control group (no intervention). We created the randomization sequence using Stata 15.0 (StataCorp, College Station, TX) statistical software.

The 25 study villages are located in Congo's North Kivu province. Humanitarian actors have been present in the province for over two decades in response to continued armed conflict, forced migration, and infectious disease epidemics. At the time of the study, an estimated 120 armed groups were active in the region (18). Conflicts between these groups, or attacks by these groups on civilians (e.g. burning villages), or the threat thereof, are the root cause for the displacements to which RRMP responds. The long-running insecurity, coupled with a lack of state investment, produces high levels of poverty. Household survey data from 2017 to 2018 indicated that in North Kivu 15% of children under 5 years old had diarrhea in the prior 2 weeks, 19% suffered from fever, and 22% from cough. Nearly 40% of mothers reported no schooling, and only 28% had completed more than primary school. Only 40% of households had access to electricity (19).

## Procedures

After beneficiary selection, RRMP publicly posted lists in each study village of those households eligible for EHI vouchers (those above the cut-off and the treatment households in our study sample). The female head of each treatment household received the vouchers at an EHI fair organized by RRMP, which took place 1 to 3 days after the lists were posted. The vouchers were distributed in detachable paper booklets with values ranging from US${\$}$0.50 to US${\$}$15, totaling US${\$}$55 to US${\$}$90; the amount varied by site and household size. Given that the prevailing wage for a day's labor reported in our focus groups was US${\$}$1, these transfers were large.

EHI fairs were temporary, one-off markets with 40 to 80 local vendors. Before the fair, RRMP provided vendors with a list of the types of preferred EHI. RRMP, together with representatives of the beneficiaries and vendors, set price ceilings. Some items were not permitted at the fair (e.g. food, livestock, medicines, and weapons). Access to the fair was restricted to voucher recipients.

The research team was completely separate from RRMP implementation. Furthermore, research assistants who interviewed study participants at baseline, 6-week follow-up, and 1-year follow-up, were blinded to households’ treatment status. They were not blinded to treatment status for the voucher use survey, which was undertaken to assess what items treatment households bought at fairs.

## Outcomes

The study's primary outcome is psychological well-being. We also assessed three possible pathways through which vouchers may impact psychological well-being: child health, social cohesion, and economic resilience. We used multiple measures for each of these outcomes (see Supplementary Material Appendix, Table S1 for variable definitions).

For psychological well-being, we used three cross-culturally validated instruments. First, we used 23 of the 25-item Hopkins Symptom Checklist (HSCL) (The default checklist contains 25 questions. We did not ask about two items, “Feeling blue”, and “Thought of ending your life”; the first could not be unambiguously translated, and the second was deemed to cause undue stress.) for anxiety and depression, regularly used in humanitarian contexts (20), including in the DRC (21, 22). For each item, like “Suddenly scared for no reason,” or “Trembling,” we asked how often the respondent had experienced such events in the preceding 2 weeks. Second, we used the World Health Organization's five-item Well-Being Index (WHO-5), which consists of simple, noninvasive, and positively worded questions. Previous studies have found that this measure has strong validity as a screening tool for depression and as an outcome measure in clinical trials, across a wide range of contexts (23). Third, we asked respondents the World Value Survey's life satisfaction question, “All things considered, how satisfied are you with your life as a whole these days on a scale of 1 to 10?.”

For child health, we collected eight measures. We asked respondents about diarrhea, cough, and fever in the previous 2 weeks for children under 5 years old, following typical DHS-style questions (24). In addition, local nurses, recruited and trained by the research team, measured children's weight, height, and mid-upper arm circumference (MUAC), to create three standard z-score indicators for malnutrition: weight-for-height, height-for-age, and MUAC-for-age (25). Nurses also administered finger or heel pricks for rapid diagnostic tests for malaria and to measure hemoglobin levels (grams per deciliter). Children that tested positive were referred to the nearby health care facility where they could be treated free of charge.

For social cohesion, we asked about household membership in village associations, requests for contributions (of labor or money) to the village in the prior 2 weeks, and thefts from the household in the prior month. We also asked about levels of trust in (1) family members, (2) another family in the village, and (3) an IDP family in the village to go to the market on behalf of the respondent.

For household resilience, we asked about household savings and debt, and income in the preceding 4 weeks. We also created a household asset index based on the ownership of 19 different items. We asked eleven standard food security questions about how many days in the preceding week certain conditions held, such as “A household member had to gather wild food,” “A household member had to hunt or harvest immature crops because of food shortage,” and so on. We also measured the household's dietary diversity, asking how many times in the past week 10 different food items were consumed. Finally, we asked about the use of alcohol or tobacco in the preceding week, and whether school-aged children were attending school or not.

## Statistical Analysis

We used intention-to-treat analyses to test for differences in outcome measures between the voucher group and the control group. We reported treatment effects for all individual outcome measures, and calculated a summary index of each of the four outcome families to avoid over-rejection of the null hypothesis due to multiple inference. To generate a summary index, we rescale each outcome so that higher values imply better outcomes, and take the average of standardized values relative to the control group (26). Treatment effects are estimated as the difference in the summary index between treatment and control groups; treatment effects are, thus expressed in standard deviation units (SDUs) relative to the control group. We estimated effects in the short (6-week follow-up) and longer term (1-year follow-up), respectively, using least squares models with fixed effects for randomization strata (villages). With the exception of hemoglobin and malaria, all variables were also measured before program onset, and we include those values in the regression model to improve statistical precision. We adjusted *P*-values for multiple hypothesis testing using step-down resampling by summary index and survey round (27). All analyses were done in Stata (version 15.0).

The study was preregistered in the EGAP registry: https://osf.io/2faj4 (short-term effects) and https://osf.io/dyb9g (longer-term effects). Deviations from the preregistration plans can be found in the Supplementary Material Appendix. Data are publicly available (https://dataverse.harvard.edu/dataset.xhtml?persistentId=doi:10.7910/DVN/OPEWXV).

## Results

### Implementation

We collaborated with RRMP on interventions implemented between August 2017 and March 2018 (see Supplementary Material Appendix, Table S2 for the implementation schedule). In total, RRMP carried out seven EHI voucher interventions in the North Kivu province, covering 25 villages. Of the 21,448 households interviewed by RRMP staff, we targeted 976 for this study (see consort diagram in Supplementary Material Appendix, Figure S1), of which 488 households were randomly assigned to the control group, and 488 to the voucher group.

### Baseline conditions and balance

For the baseline survey, we successfully identified and interviewed 856 (88%) of the targeted households (424 from the treatment group and 432 from the control group). For the voucher use survey, conducted 3 to 8 days after the fair, we interviewed 434 treatment households (89% of households assigned to voucher group). Loss to follow-up was 10% after 6 weeks (769 surveys conducted successfully) and 25% after 1 year (643 surveys). Loss to follow-up was not associated with treatment assignment at baseline, 6-week follow-up, or 1-year follow-up (Supplementary Material Appendix, Table S3).

Prior to voucher distribution, there were no systematic differences by treatment status (Supplementary Material Appendix, Table S4). Respondents were 35 years old, on average, lived with 5.5 other household members, and 88% were female. A total of 22% of respondents were born in the hosting village. Among those not born in the village, 75% of respondents had arrived in the village less than 12 months prior to the intervention, and 87% had arrived in the last 5 years.

The mean HSCL anxiety/depression score was 1.51; 284 respondents (33%) had a score over the commonly used cutoff (> 1.75) that indicates clinically significant anxiety or depression (21). The mean WHO-5 Well-Being score was 0.96 (1 = “Some or little of the time”) out of four (0 = “Not at all”; 3 = “Most or all of the time”), and mean life satisfaction was low, at 3.1 out of 10.

In terms of child health, 31% of children under 5 years old had diarrhea in the prior 2 weeks, 56% fever, and 48% cough.

Respondents were members of 0.58 associations on average. A total of 249 respondents (29%) had been asked to contribute to the village in the previous 2 weeks, and 228 (27%) reported that something had been stolen from their household in the last month. The mean reported trust in family, IDPs, or other families in the village to go to the market on the respondent's behalf was 3.7 (3 = “Neither trust nor distrust”; 4 = “Completely trust”).

Households owned 20.4 asset items (across 19 asset categories), on average, with the most common items being clothing and pots. Only 7% of households owned a radio. Mean household income was US${\$}$12 in the month prior to the survey, and households had US${\$}$6.4 in savings and US${\$}$18 in debt. The mean food insecurity score was 2.10, meaning that the typical household undertook an activity in response to insufficient food (e.g. skipping meals) more than 2 days out of the previous week. The mean dietary diversity score was 1.98; i.e. households consumed foods in each of 10 categories just under 2 days in the previous week. Households consumed alcohol or tobacco 0.43 days in the past week. Just under half (49.5%) of children 5 to 18 years old were in school.

Across 20 focus group discussions, which we organized before and after the program to learn more about the displacement dynamics, many participants suggested that they expect that the government will tell them when it is safe to return, and that it will be safe to return when government soldiers have secured the area. Other participants suggested that before returning they first needed to observe the security situation first-hand, for example during brief return trips to their home fields.

### Use of EHI vouchers

At the fairs, treatment households used EHI vouchers to purchase clothes (86% of treatment households), cloth (74%), pots and pans (56%), soap (51%), mattresses (35%), blankets (33%), luggage (27%), and buckets and basins (27%; see Supplementary Material Appendix, Table S5). Conditional on purchasing an item, voucher recipients spent the largest amounts on the following items: clothes (US${\$}$20.17), cloth (US${\$}$17.64), mattresses (US${\$}$27.99), blankets (US${\$}$13.00), chairs, beds, or tables (US${\$}$11.50), and tarp (US${\$}$17.39; Supplementary Material Appendix, Table S5).

### Psychological well-being

**Fig. 1.**
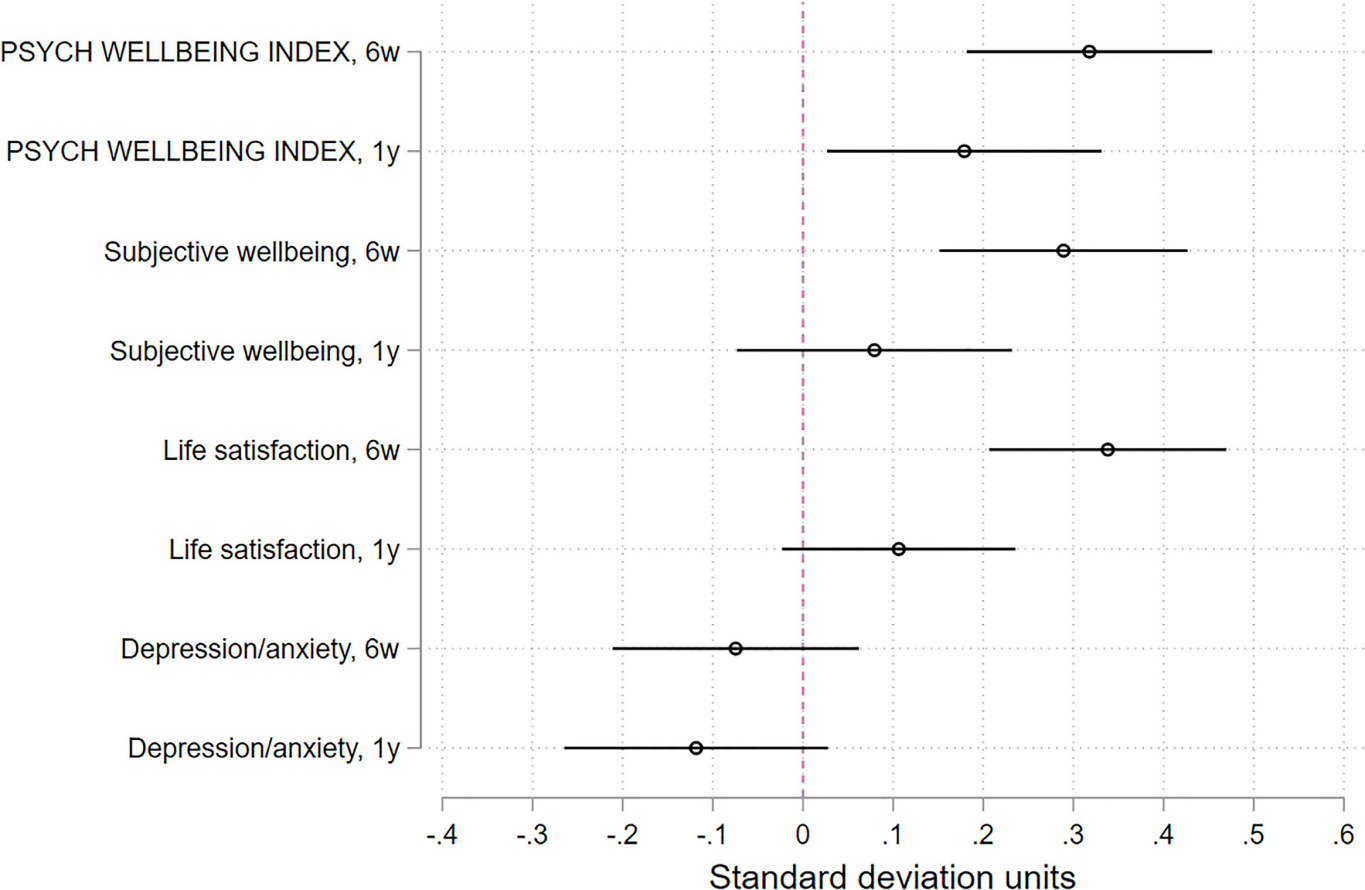
Average treatment effect of vouchers on the psychological well-being index, and its three components: subjective well-being, life satisfaction, and depression/anxiety. Higher values indicate improvements, with the exception of depression/anxiety, where lower values indicate improvements. Bars show 95% confidence intervals. Intention-to-treat estimates from least squares models with fixed effects for randomization strata (25 villages).

We find positive and large treatment effects on the psychological well-being summary index at 6 weeks (mean index difference 0.32 SDUs [95% CI 0.18 to 0.45]) and at 1 year (mean index difference 0.18 SDUs [95% CI 0.03 to 0.33]; Figure 1; Supplementary Material Appendix, Table S6). At 6 weeks, the entire distribution of psychological well-being shifted right (improved) in the treatment group. At 1 year, the shift is similar, but less pronounced, corresponding to a smaller treatment effect (Supplementary Material Appendix, Figures S2 and S3). For individual measures at 6 weeks, the WHO-5 Well-being index is 1.09 for control households and 1.29 for treatment households; an improvement of 19% (*P-adj* < 0.01). Life satisfaction is 3.29 in the control group and 3.88 in the treatment group; an 18% increase (*P-adj* < 0.01). The anxiety/depression score (HPCL) decreased from 1.38 to 1.33, although this improvement is not statistically significant. After 1 year, although differences in individual measures were no longer statistically significant, all three point estimates suggest beneficial treatment effects. In sum, the positive treatment effects suggest that even modest economic transfers can improve recipient psychological well-being in both the short and longer run.

## Child Health, Social Cohesion, and Resilience

**Fig. 2.**
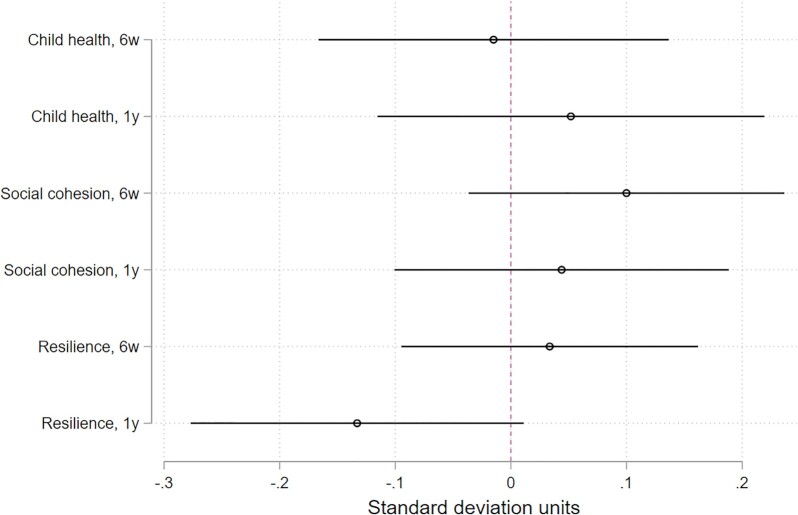
Average treatment effect of vouchers on indices of physical health, social cohesion, and resilience, at 6 weeks and 1 year after voucher distribution. Bars show 95% confidence intervals. Intention-to-treat estimates from least squares models with fixed effects for randomization strata (25 villages).

We find no treatment effects on child health at 6 weeks (mean index difference −0.02 SDUs) or at 1 year (mean index difference 0.05 SDUs; see Figure 2). The proportion of children in a household with diarrhea, cough, or fever is statistically equivalent between treatment and control households at 6 weeks and 1 year (Supplementary Material Appendix, Table S6). The same holds for length-for-age, weight-for-height, and MUAC-for-age z-scores, as well as hemoglobin levels and malaria. Repeating this analysis at the child instead of household level, yields similar results (Supplementary Material Appendix, Table S7). This implies that larger or different transfers may be needed to improve child health.

There is no evidence for treatment effects on social cohesion at 6 weeks (mean index difference 0.10 SDUs) or at 1 year (mean index difference 0.01 SDUs; see Figure 2). (SI Appendix Table S8 shows that these results hold for households born inside and outside the village.) That is, at both time points, there are no statistically significant differences between treatment and control households in terms of group membership, requests for contributions to the village, trust, or theft (Supplementary Material Appendix, Table S6). These findings suggest that targeting some households and not others within the same community did not undermine local community relations, and implies that this type of aid may fit the do no harm principle of humanitarian assistance.

Finally, we find no overall treatment effect on resilience at 6 weeks (mean index difference 0.03 SDUs), nor at 1 year (mean index difference −0.13 SDUs). (When debt and the use of alcohol or tobacco (see the discussion below) enter the family outcome as contributing to rather than undermining resilience, the mean effect is positive and statistically significant at six weeks: mean index difference 0.27 SDUs, *P* < 0.01. At one year, the mean index difference is 0.04 SDUs (*P*> 0.1).) However, the data suggest some important changes in several index components in the short run. At 6 weeks, treatment households’ asset index improved by 13.3% (1.36 vs. 1.20; mean difference 0.16, *P*-adj < 0.01; Supplementary Material Appendix, Table S6), and dietary diversity increased from 2.15 to 2.28 (mean difference 0.13, *P*-adj < 0.1). Furthermore, and contrary to our preanalysis expectations (28), we also find that treatment households have 43% more debt (US${\$}$23.24 vs. US${\$}$16.27; mean difference US${\$}$6.97, *P*-adj < 0.05), and consumed more alcohol or tobacco: from 0.26 days to 0.46 days per week (mean difference 0.2, *P*-adj < 0.01). (At one year, treatment households reported using alcohol or tobacco 0.55 days per week, compared to 0.29 days for control households (mean difference 0.26).) These findings are consistent with multiple possible mechanisms. On the one hand, treatment households with more assets may have become more credit worthy (because they own more collateral), or they may have increased expenditure on consumption goods. On the other hand, voucher recipients may have felt compelled to take on debt of those without assistance. Similarly, treatment households may use tobacco and alcohol to cope with the stress of displacement. On the other hand, they may have increased consumption to create and strengthen social bonds.

For the RRMP funding year that we study (2017/2018), UNICEF estimated that US${\$}$3,918,388 was transferred to 269,677 beneficiaries via EHI fairs, or US${\$}$14.53 per beneficiary. This excludes implementation costs, which are estimated to be US${\$}$1,713,204. Estimated total cost per beneficiary (this includes all members of a transfer recipient household), is thus US${\$}$20.88. An earlier study in an IDP camp in eastern DRC found costs, excluding the transfer amount, of US${\$}$14.35 per food voucher household and US${\$}$11.34 per cash transfer household (28). If we exclude the transfer amount, and assume six people per household, we estimate a cost of US${\$}$38 per household. The higher cost we find here is likely the result of the logistical costs to rapidly reach displaced people living in rural host communities, compared to slower assistance to camps as in (28).

## Discussion and Implications for Policy

We find that a low-cost, unconditional, economic transfer program has large positive impacts on psychological well-being of vulnerable and displaced persons fleeing armed conflict in both the short and longer run. Thus, humanitarian aid delivered soon after displacement events has the potential to significantly improve recipient well-being. We find no evidence that the program undermined social cohesion in the village, a potential concern with aid programs that target some village residents but not others, particularly in settings where nearly all households are vulnerable. The lack of evidence for improvements in child health suggests the need for larger or different types of transfers, as the challenges to health are pervasive in these contexts, given the high incidence of malaria, pneumonia, diarrhea and other diseases.

The results related to psychological well-being may be influenced by spillovers. That is, the improvement in psychological well-being may be driven not by improvements among treated households, but by a worsening in the psychological well-being among the control households caused by the treatment. Our design allows us to test this claim. In Congo, many IDP families are hosted by other families, and thus a dwelling may contain multiple households. As part of our data collection, whenever this was the case, we collected data from the household that was assigned treatment or control status by the lottery, plus the other household in that dwelling. Thus, we can learn about spillovers within the dwelling experimentally by comparing the effect of economic assistance on households that share a dwelling with treatment households to those that share a dwelling with control households. We find no difference in psychological well-being between these two groups, although households sharing a dwelling with treatment households do report higher life satisfaction (Supplementary Material Appendix, Table S9). The positive impact on psychological well-being is thus unlikely to result from treatment lowering psychological well-being in the control group.

Another worry may be that the results related to psychological well-being are influenced by experimenter demand effects. We think this is unlikely for several reasons. First, the research team was completely separate from program implementation, and research assistants were blinded to respondents’ treatment status. Second, the individual variables that constitute the psychological well-being index all point in the same direction (Supplementary Material Appendix, Table S6), although they differ in question construction and dimensions of psychological well-being that they aim to measure. Third, the psychological well-being measures that we make use of have been extensively validated, including in the DRC (20–23). Finally, we do not find treatment effects on the other outcome indices, and specifically social cohesion and resilience, which also include measures susceptible to experimenter demand effects.

Our study population consists predominantly of subsistence farmers living in chronic poverty and insecurity with little access to markets and public services. There are hundreds of millions of people living in similar conditions around the world, including in places like Yemen, South Sudan, Nigeria, and Afghanistan (29). Lessons from our study are thus likely relevant for other programs that aim to improve the well-being of other vulnerable populations in fragile contexts.

There is still much to learn about the consequences of forced displacement and how aid programs can best function in displaced populations. In the process of displacement, households must leave behind their house and most of their belongings, and often have traumatic experiences, all reducing their ability to cope. This may result in a vicious psychological poverty trap, as poor mental health undercuts productive investments. We show that access to essential resources helps improve psychological well-being. Therefore, providing short run emergency relief may reverse a psychological poverty trap and stimulate longer-run development (5, 6, 30).

It is an open question how the mental health effects of economic transfers compare to direct mental health programs, and whether economic transfers are more effective if they are combined with mental health programming (see (31) for evidence from conflict-affected youth in Liberia, and (32) for evidence from Kenya. However, these are not humanitarian assistance programs.). It is possible that some mental disorders are unresponsive to economic transfers and require specialized psychiatric care. Yet, economic transfers may still be able to ameliorate a significant fraction of the burden of mental disorder. Future work should delineate the combinations of disorders, contexts, and interventions that maximize welfare.

## Supplementary Material

pgac101_Supplemental_Files
