# Supplementary material for: Cash-like vouchers improve psychological well-being of vulnerable and displaced persons fleeing armed conflict
Source: PNAS Nexus. 2022 Jun 30;1(3):pgac101. doi: 10.1093/pnasnexus/pgac101 (PMC9896939; doi:10.1093/pnasnexus/pgac101)
Supplement: pgac101_Supplemental_Files [file pgac101_supplemental_files.zip › PNASNEXUS-PNASNEXUS-2022-00251-T-s02.docx]

Supporting information appendix

Figure S1. Trial profile

**Figure S2. Distribution of psychological well-being at six weeks after voucher distribution**


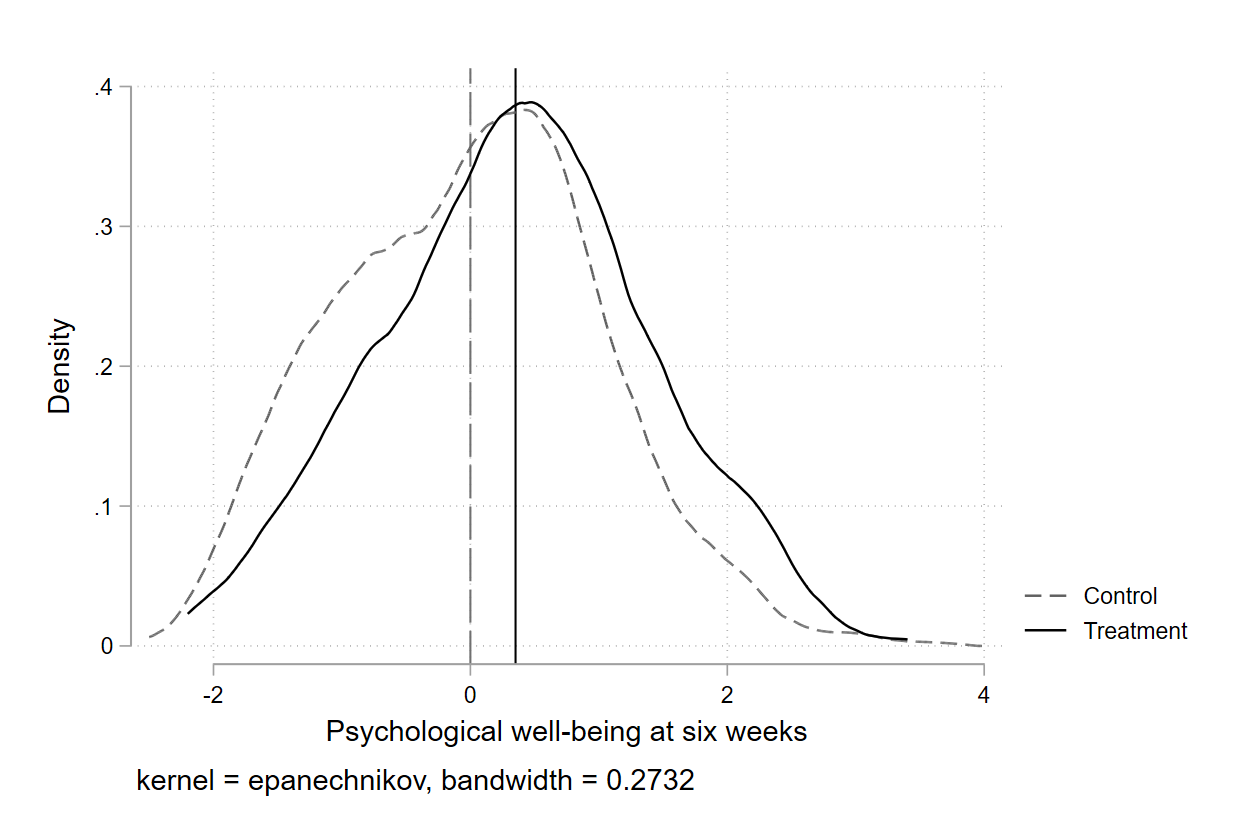


**Figure S3. Distribution of psychological well-being at one year after voucher distribution**

**
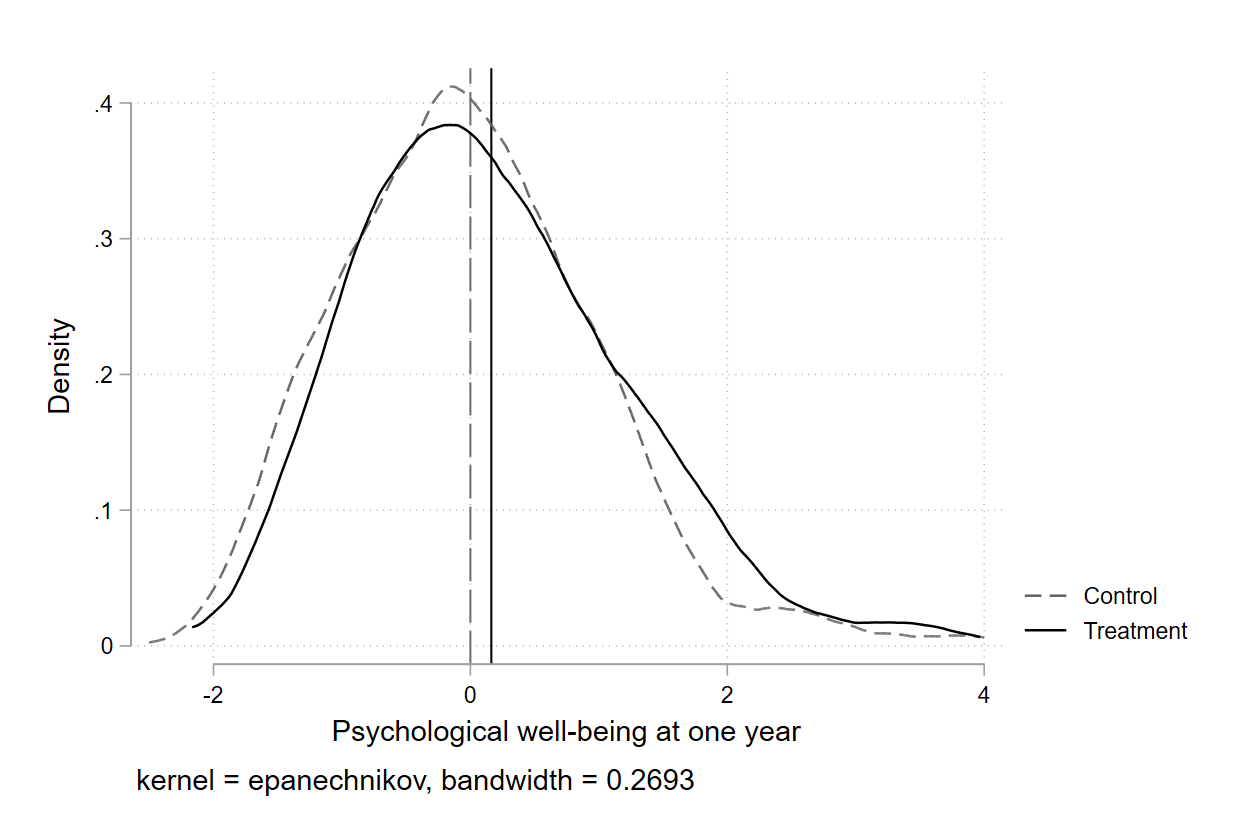
**

Table S1. Variable definitions

| **Family** | **Outcome** | **Description** | **BL** | **6w** | **1y** |
| --- | --- | --- | --- | --- | --- |
| Psychological well-being | Anxiety / depression | Modified version of Hopkins Symptom Checklist (HSCL). Continuous 0 to 3. Average across 23 statements. Over the last two weeks, have you experienced: 1) Suddenly scared for no reason, 2) Feeling fearful, 3) Faintness, dizziness or weakness, 4) Nervousness or shakiness inside, 5) Heart pounding or racing, 6) Trembling, 7) Feeling tense or keyed up, 8) Headache, 9) Spell of terror or panic, 10) Feeling restless or can’t sit still, 11) Feeling low in energy, slowed down, 12) Blaming yourself for things, 13) Crying easily, 14) Loss of sexual interest or pleasure, 15) Poor appetite, 16) Difficulty falling asleep, staying asleep, 17) Feeling hopeless about future, 18) Feeling lonely, 19) Feeling of being trapped or caught, 20) Worry too much about things, 21) Feeling no interest in things, 22) Feeling everything is an effort, 23) Feeling of worthlessness. Response options are: 0) Not at all, 1) Some or little of the time, 2) Occasionally or a moderate amount of time, 3) Most or all the time. | 1 | 1 | 1 |
| Psychological well-being | Well-being | WHO-5 well-being index. Continuous 0 to 3. Average across the following statements. Over the last two weeks: 1) I have felt cheerful and in good spirits, 2) I have felt calm and relaxed, 3) I have felt active and vigorous, 4) I woke up feeling fresh and rested, 5) My daily life has been filled with things that interest me. Response options: 0) Not at all, 1) Some or little of the time, 2) Occasionally or a moderate amount of time, 3) Most or all the time. | 1 | 1 | 1 |
| Psychological well-being | Life satisfaction | World Value Survey life satisfaction measure. Continuous 1 to 10. Response to “All things considered, how satisfied are you with your life as a whole these days on a scale of 1 to 10?” 1= very dissatisfied, and 10= very satisfied. | 1 | 1 | 1 |
| Child Health | Diarrhea | Continuous 0 to 1. Share of children (under 5 years of age) that had diarrhea in the last two weeks. As reported by the respondent. | 1 | 1 | 1 |
| Child Health | Fever | Continuous 0 to 1. Share of children (under 5 years of age) that had fever in the last two weeks. As reported by the respondent. | 1 | 1 | 1 |
| Child Health | Cough | Continuous 0 to 1. Share of children (under 5 years of age) that had a cough in the last two weeks. As reported by the respondent. | 1 | 1 | 1 |
| Child Health | Weight to height z-score | Continuous. For all children under the age of five years, measured their weight and height, and then compute weight as a z-score of the WHO’s average measures for children of the same height. | 0 | 1 | 1 |
| Child Health | Height to age z-score | Continuous. For all children under the age of five years, measured their height and then compute it as a z-score of the WHO’s average measures for children of the same age in months. | 0 | 1 | 1 |
| Child Health | MUAC to age z-score | Continuous. For all children under the age of five years, measured their mid upper arm circumference (MUAC) and then compute it as a z-score of the WHO’s average measures for children of the same age in months. | 0 | 1 | 1 |
| Child Health | Hemoglobin | Continuous in grams per decilitre (g/dL). Child’s hemoglobin level as measured in blood sample. | 0 | 1 | 1 |
| Child Health | Malaria | Binary. Positive or negative result of malaria Rapid Diagnostic Test. | 0 | 1 | 1 |
| Social Cohesion | Membership | Continuous 0 to 11. Number of associations the household is a member of: 1) Credit or savings, 2) Farming, 3) Protection/ security, 4) Women, 5) Youth, 6) Religious, 7) Conflict resolution, 8) Development, 9) Health, 10) Education, 11) Other | 1 | 1 | 1 |
| Social Cohesion | Contributions | Binary. In the last two weeks, have you been asked to contribute to the village? Yes=1, No=0 | 1 | 1 | 1 |
| Social Cohesion | Trust | Continuous 1 to 5. Average across the following. How much would you trust: 1) family members, 2) another family in the village, and 3) an IDP family in the village to go to the market for you if you can't go yourself? Response options: 1) Completely distrust, 2) Somewhat distrust, 3) Neither trust nor distrust, 4) Somewhat trust, 5) Completely trust | 1 | 1 | 1 |
| Social Cohesion | Theft | Binary. Has anything been stolen from your household in the past month? | 1 | 1 | 1 |
| Resilience | Assets | Continuous. Average number of items owned of the following list: identity card, chair, bicycle, motorcycle, hoe, cloth, generator (for electricity), flashlight, radio, mattress, blankets, jerry can, bed net (treated or not), tarp, clothes other, soap, buckets, pots and pans, luggage. | 1 | 1 | 1 |
| Resilience | Savings | Continuous in US dollars. How much does your household have in savings? | 1 | 1 | 1 |
| Resilience | Income | Continuous in US dollars. In the last 4 weeks, how much income did your household earn or receive? (Through labor, sales, remittances, etc.) | 1 | 1 | 1 |
| Resilience | Dietary diversity | Continuous 0 to 7. Average across the following: In the last seven days, how many days has your household eaten or consumed: 1) Corn, sorghum, rice, bread, 2) Cassava, plantains, other tubers, 3) Peanuts, beans, peas, lentils, etc., 4) Vegetables (and their leaves), 5) Fruits, 6) Meat, fish, chicken, eggs, 7) Milk, cheese, yogurt, other dairy, 8) Sugar, honey, other sweeteners, 9) Oils and fats, 10) Condiments, spices. | 1 | 1 | 1 |
| Resilience | Food insecurity | Continuous 0 to 7. Average across the following: In the last seven days, how many times: 1) Have adults cut the size of meals or skipped meals?, 2) Have adults gone a whole day without meals?, 3) Have children (<14yo) cut the size of meals or skipped meals?, 4) Have children (<14yo) gone a whole day without meals?, 5) Have household members had to eat less preferred or less expensive foods?, 6) Have household members had to borrow food or rely on help from a friend or relative to get enough food?, 7) Have household members had to purchase food on credit?, 8) Have household members had to gather wild food, hunt, or harvest immature crops because of food shortage?, 9) Have household members had to consume seed stock held for next season?, 10) Have household members had to go elsewhere to eat because there was not enough food in the house?, 11) Have household members had to go beg because there was not enough food in the house? | 1 | 1 | 1 |
| Resilience | Kids in school | Continuous 0 to 1. Proportion of children aged 5-18 in school per household. | 1 | 1 | 1 |
| Resilience | Debt | Continuous in US dollars. How much does your household owe in debts? | 1 | 1 | 1 |
| Resilience | Alcohol, Tobacco | Continuous 0 to 7. In the last seven days, how many days has your household consumed alcohol or tobacco. | 1 | 1 | 1 |

Notes: Data collected during “BL”= Baseline survey; “6w” = six week follow-up; “1y” = one year follow-up.

Table S2. Data collection sites and dates

| # | Site | Territory | #Vills. | | Baseline | Voucher use survey | Short term | Longer term |
| --- | --- | --- | --- | --- | --- | --- | --- | --- |
| 1 | Butale | Masisi | 2 | Aug 9-12, 2017 | | Aug 12-16, 2017 | Sep 13-18, 2017 | Sep 18-23, 2018 |
| 2 | Kibarizo | Masisi | 3 | Sep 8-13, 2017 | | Sep 13-16, 2017 | Oct 20-26, 2017 | Sep18-23, 2018 |
| 3 | Kitsombiro | Lubero | 3 | Nov 21-26, 2017 | | Nov 30-Dec 12, 2017 | Jan 11-17, 2018 | Jun 25-30, 2019 |
| 4 | Mbau | Beni | 2 | Dec 7-12, 2017 | | Dec 12-15, 2017 | Jan 23-28, 2018 | Sept 19-24, 2019 |
| 5 | Kirumbu | Masisi | 4 | Jan 27-Feb 2, 2018 | | Feb 2-7, 2018 | Mar 14-19, 2018 | Dec 13-20, 2018 |
| 6 | Pinga | Walikale | 3 | Feb 7-16, 2018 | | Feb 19 -23, 2018 | Mar 29-Apr 9, 2018 | Apr13-26, 2019 |
| 7 | Nyabiondo | Masisi | 8 | Mar 30-Apr 4, 2018 | | Apr 5-7, 2018 | May 19-27, 2018 | Feb28-Mar 7, 2019 |

Notes: # Vills. = research villages in the intervention site, which are also our randomization strata. The initial longer-term visits to Sites 1 and 2 lacked migration modules, so those sites were re-visited between November 7-10, 2018, to conduct the migration modules.

Table S3. Attrition

|  | Target | Number  interviewed | Missing from treatment | Missing from control | Association between treatment and attrition | Standard error |
| --- | --- | --- | --- | --- | --- | --- |
| Baseline | 976 | 856 | 64 | 56 | 0.02 | (0.02) |
| Six weeks after baseline | 976 | 769 | 107 | 100 | 0.01 | (0.03) |
| One year after baseline | 976 | 643 | 167 | 166 | -0.02 | (0.03) |

Notes: Target refers to the number of households that participated in the lottery. The association between treatment (voucher) and attrition was estimated with a linear model of attrition as a function of treatment assignment, controlling for randomization strata. In fact, 690 surveys were conducted one year after baseline, however only for 643 of these did we also collect data at baseline.

**Table S4. Balance table of baseline characteristics**

|  | Control group  (n=432) | | Treatment group  (n=424) | |
| --- | --- | --- | --- | --- |
|  | Mean or n | sd or % | Mean or n | sd or % |
| Age of respondent | 36.44 | 14.49 | 34.77 | 13.08 |
| Household size | 6.49 | 2.42 | 6.60 | 2.68 |
| Female respondent | 381/432 | 88% | 371/424 | 88% |
| Born in the village | 0.78 | 0.41 | 0.78 | 0.41 |
| - If not, arrived less than 12 months prior | 0.77 | 0.42 | 0.75 | 0.43 |
| - If not, arrived less than five years prior | 0.86 | 0.34 | 0.89 | 0.32 |
| Anxiety/depression score (0-3) | 1.53 | 0.55 | 1.50 | 0.58 |
| Well-being score (0-3) | 0.93 | 0.60 | 0.99 | 0.62 |
| Life satisfaction score (1-10) | 3.07 | 1.67 | 3.17 | 1.71 |
| Diarrhea in children <5y in last 2 weeks | 209/673 | 31% | 194/657 | 30% |
| Fever in children <5y in last 2 weeks | 371/671 | 55% | 371/654 | 57% |
| Cough in children <5y in last 2 weeks | 304/672 | 45% | 337/657 | 51% |
| Associations in which household is a member (0-11) | 0.55 | 0.78 | 0.60 | 0.84 |
| Requests for contributions to the village in last two weeks | 0.25 | 0.43 | 0.33 | 0.47 |
| Trust in village score (1-5) | 3.69 | 0.84 | 3.71 | 0.87 |
| Had something stolen form household in last month | 0.27 | 0.44 | 0.26 | 0.44 |
| Household assets (mean across 20 items) | 1.15 | 0.71 | 1.17 | 0.67 |
| Household income in last month (US$) | 12.88 | 19.32 | 11.79 | 14.05 |
| Household savings (US$) | 5.27 | 44.19 | 7.60 | 59.46 |
| Household debt (US$) | 20.75 | 50.29 | 15.28 | 26.03 |
| Dietary diversity score (0-7) | 2.00 | 0.81 | 1.96 | 0.78 |
| Food insecurity score (0-7) | 2.14 | 0.94 | 2.08 | 0.90 |
| Days consumed alcohol or tobacco in last week (0-7) | 0.46 | 1.42 | 0.40 | 1.15 |
| Proportion of children 5-18 in school | 0.47 | 0.39 | 0.46 | 0.41 |

Notes: See Table S1 for variable definitions. Measures of diarrhea, fever and cough are for children under 5 years old, rather than for each respondent.

Table S5. Voucher spending at EHI fairs

| EHI | Observations | Share of household that purchased item | Average expenditure by those households that purchased the item (US$) |
| --- | --- | --- | --- |
| Clothes | 427 | 0.86 | 20.17 |
| Cloth | 427 | 0.74 | 17.64 |
| Pots and pans | 427 | 0.56 | 9.53 |
| Soap | 426 | 0.51 | 2.33 |
| Mattress | 424 | 0.35 | 27.99 |
| Blanket | 427 | 0.33 | 13.00 |
| Buckets and basins | 426 | 0.27 | 4.00 |
| Luggage | 427 | 0.27 | 14.14 |
| Tarp | 427 | 0.17 | 17.39 |
| Radio | 426 | 0.15 | 11.37 |
| Flash light | 427 | 0.11 | 4.57 |
| Jerry can | 427 | 0.10 | 3.10 |
| Farming tools | 426 | 0.04 | 4.18 |
| Generator | 425 | 0.01 | 14.67 |
| Bed net | 427 | 0.01 | 1.25 |
| Chairs, beds or tables | 427 | 0.01 | 11.50 |
| Other items | 419 | 0.69 | 13.53 |

Notes: Summary information from 434 interviews with voucher recipients 1-3 days after the EHI fair. “Other items” includes plates, bowls, jugs, footwear, bedsheets, thermoses, batteries, and solar panels.

Table S6. Effect of vouchers on psychological well-being, child health, social cohesion, and resilience

|  | Six week follow up | | | |  |  | |  | |  | | One year follow up | | | | |  |  | |  | |  | | |
| --- | --- | --- | --- | --- | --- | --- | --- | --- | --- | --- | --- | --- | --- | --- | --- | --- | --- | --- | --- | --- | --- | --- | --- | --- |
|  |  |  | 95% CI | |  | |  | |  | |  | |  | 95% CI | |  | | |  | |  | | |  |
|  | Control | T-C | Lower | Upper | p | | p-adj. | | N | | Control | | T-C | Lower | Upper | p | | | p-adj. | | N | |  |  |
| *Psychological well-being* | 0.00 | 0.32 | 0.18 | 0.45 | 0.00 | | 0.00 | | 769 | | -0.02 | | 0.18 | 0.03 | 0.33 | 0.02 | | | 0.07 | | 643 | |  |  |
| Anxiety/depression | 1.38 | -0.05 | -0.13 | 0.04 | 0.29 | | 0.27 | | 769 | | 1.48 | | -0.07 | -0.16 | 0.02 | 0.11 | | | 0.27 | | 643 | |  |  |
| Well-being | 1.09 | 0.20 | 0.10 | 0.29 | 0.00 | | 0.00 | | 769 | | 1.09 | | 0.06 | -0.05 | 0.17 | 0.31 | | | 0.30 | | 642 | |  |  |
| Life satisfaction | 3.29 | 0.59 | 0.36 | 0.82 | 0.00 | | 0.00 | | 769 | | 3.06 | | 0.17 | -0.04 | 0.39 | 0.11 | | | 0.27 | | 633 | |  |  |
| *Child health* | 0.00 | -0.02 | -0.17 | 0.14 | 0.85 | | 0.85 | | 606 | | 0.00 | | 0.05 | -0.12 | 0.22 | 0.54 | | | 0.78 | | 506 | |  |  |
| Diarrhea | 0.30 | 0.01 | -0.05 | 0.08 | 0.73 | | 0.98 | | 512 | | 0.27 | | 0.02 | -0.06 | 0.10 | 0.65 | | | 0.97 | | 382 | |  |  |
| Fever | 0.57 | -0.04 | -0.11 | 0.03 | 0.30 | | 0.81 | | 511 | | 0.44 | | 0.03 | -0.06 | 0.12 | 0.47 | | | 0.97 | | 380 | |  |  |
| Cough | 0.48 | 0.01 | -0.07 | 0.08 | 0.88 | | 0.98 | | 511 | | 0.41 | | 0.01 | -0.08 | 0.10 | 0.86 | | | 0.97 | | 381 | |  |  |
| Weight-for-height | 0.26 | 0.17 | -0.03 | 0.37 | 0.09 | | 0.54 | | 511 | | 0.41 | | 0.15 | -0.08 | 0.39 | 0.20 | | | 0.97 | | 460 | |  |  |
| Height-for-age | -2.46 | -0.04 | -0.30 | 0.23 | 0.77 | | 0.98 | | 505 | | -2.63 | | 0.06 | -0.22 | 0.34 | 0.66 | | | 0.80 | | 463 | |  |  |
| MUAC-for-age | -0.34 | -0.07 | -0.23 | 0.09 | 0.39 | | 0.86 | | 507 | | -0.61 | | 0.15 | -0.02 | 0.32 | 0.09 | | | 0.97 | | 467 | |  |  |
| Hemoglobin | 10.95 | -0.12 | -0.31 | 0.07 | 0.20 | | 0.72 | | 506 | | 10.45 | | 0.08 | -0.14 | 0.29 | 0.49 | | | 0.54 | | 453 | |  |  |
| Malaria | 0.10 | -0.03 | -0.07 | 0.01 | 0.14 | | 0.65 | | 509 | | 0.08 | | -0.02 | -0.06 | 0.01 | 0.23 | | | 0.97 | | 456 | |  |  |
| *Social cohesion* | 0.00 | 0.10 | -0.04 | 0.24 | 0.15 | | 0.36 | | 769 | | 0.01 | | 0.01 | -0.13 | 0.16 | 0.86 | | | 0.85 | | 643 | |  |  |
| Membership | 0.49 | 0.07 | -0.03 | 0.17 | 0.16 | | 0.42 | | 769 | | 0.59 | | -0.06 | -0.18 | 0.06 | 0.31 | | | 0.63 | | 643 | |  |  |
| Contributions | 0.24 | 0.08 | 0.02 | 0.14 | 0.01 | | 0.04 | | 769 | | 0.40 | | -0.02 | -0.09 | 0.05 | 0.58 | | | 0.81 | | 643 | |  |  |
| Trust | 3.83 | -0.01 | -0.12 | 0.10 | 0.87 | | 0.87 | | 768 | | 3.87 | | 0.01 | -0.11 | 0.14 | 0.81 | | | 0.82 | | 642 | |  |  |
| Theft | 0.25 | -0.02 | -0.08 | 0.04 | 0.51 | | 0.75 | | 767 | | 0.32 | | 0.06 | -0.01 | 0.13 | 0.12 | | | 0.38 | | 641 | |  |  |
| *Resilience* | 0.00 | 0.03 | -0.09 | 0.16 | 0.61 | | 0.85 | | 769 | | 0.01 | | -0.13 | -0.28 | 0.01 | 0.07 | | | 0.17 | | 643 | |  |  |
| Assets | 1.20 | 0.16 | 0.08 | 0.23 | 0.00 | | 0.00 | | 769 | | 1.40 | | 0.01 | -0.09 | 0.10 | 0.87 | | | 1.00 | | 643 | |  |  |
| Savings | 1.41 | 0.32 | -0.66 | 1.29 | 0.52 | | 0.76 | | 769 | | 7.56 | | -0.36 | -8.41 | 7.69 | 0.93 | | | 1.00 | | 643 | |  |  |
| Income | 14.24 | -0.01 | -2.45 | 2.44 | 1.00 | | 1.00 | | 769 | | 17.37 | | -0.92 | -4.15 | 2.30 | 0.57 | | | 1.00 | | 643 | |  |  |
| Dietary diversity | 2.15 | 0.13 | 0.02 | 0.23 | 0.02 | | 0.08 | | 769 | | 2.10 | | -0.03 | -0.16 | 0.10 | 0.62 | | | 1.00 | | 643 | |  |  |
| Food insecurity | 1.79 | -0.07 | -0.18 | 0.05 | 0.24 | | 0.55 | | 769 | | 1.99 | | 0.03 | -0.11 | 0.16 | 0.72 | | | 1.00 | | 643 | |  |  |
| Kids in school | 0.48 | 0.05 | 0.00 | 0.11 | 0.06 | | 0.20 | | 642 | | 0.58 | | -0.01 | -0.07 | 0.05 | 0.80 | | | 1.00 | | 530 | |  |  |
| Debt | 16.27 | 6.97 | 2.44 | 11.51 | 0.00 | | 0.01 | | 767 | | 34.81 | | 1.10 | -8.50 | 10.70 | 0.82 | | | 1.00 | | 642 | |  |  |
| Alcohol, Tobacco | 0.26 | 0.20 | 0.04 | 0.37 | 0.01 | | 0.08 | | 768 | | 0.29 | | 0.26 | 0.08 | 0.44 | 0.01 | | | 0.03 | | 641 | |  |  |

Notes: Indices are calculated by rescaling each outcome in each family (e.g. psychological well-being) so that higher values imply better outcomes, then standardizing relative to the endline control group, following Kling et al. (2007). If a household was missing an observation for one of the index components, we took the average of the remaining non-missing individual variables for that household. Treatment effects are from intention-to-treat analyses with fixed effects for each randomization stratum (25 villages) (Abadie et al 2017). For all outcome variables, except anthropometry, hemoglobin, and malaria, we control for baseline values. Treatment effects are in standard deviation units relative to the control group. “Control” column indicates average value of the dependent value in the control condition at 6 weeks and 1 year, respectively. The number of observations for child health measures are lower because these are only collected for those households with at least one child younger than five years old. The column ‘p-adj’ contains p-values adjusted for multiple hypothesis testing using the free step-down resampling methodology of Westfall and Young (1993). The mean effects estimates are adjusted for comparisons to each other, by survey round (n=4 each round). The index components are adjusted for comparisons within each family, for each survey round (e.g. n=3 for mental health at six week follow-up). See Table S1 for variable definitions. MUAC = middle upper arm circumference.

Table S7. Effect of vouchers on child health outcomes, child level analysis

| Baseline | | | | | | Six weeks | | | | | | | One year | | | | | | |
| --- | --- | --- | --- | --- | --- | --- | --- | --- | --- | --- | --- | --- | --- | --- | --- | --- | --- | --- | --- |
|  | Control | |  | Treatment |  | Control | Treatment | |  | CI 95% | |  | Control | Treatment | |  | CI 95% | |  |
| Outcomes | Mean | | n | Mean | n | Mean | Mean | T-C | | Lower | Upper | n | Mean | Mean | T-C | | Lower | Upper | n |
| Diarrhea | | 0.31 | 673.00 | 0.30 | 657 | 0.31 | 0.31 | 0.00 | | -0.06 | 0.06 | 1117 | 0.26 | 0.29 | 0.04 | | -0.03 | 0.10 | 863 |
| Fever | | 0.55 | 671.00 | 0.57 | 654 | 0.53 | 0.52 | 0.00 | | -0.07 | 0.06 | 1117 | 0.44 | 0.46 | 0.03 | | -0.05 | 0.10 | 858 |
| Cough | | 0.45 | 672.00 | 0.51 | 657 | 0.45 | 0.49 | 0.05 | | -0.01 | 0.12 | 1117 | 0.41 | 0.41 | 0.00 | | -0.07 | 0.08 | 863 |
| Weight-for-height | |  |  |  |  | 0.23 | 0.35 | 0.14 | | -0.05 | 0.33 | 920 | 0.44 | 0.50 | 0.03 | | -0.19 | 0.26 | 741 |
| Height-for-age | |  |  |  |  | -2.43 | -2.52 | -0.08 | | -0.33 | 0.18 | 885 | -2.59 | -2.56 | 0.06 | | -0.19 | 0.32 | 733 |
| MUAC-for-age | |  |  |  |  | -0.37 | -0.50 | -0.10 | | -0.25 | 0.05 | 884 | -0.61 | -0.57 | 0.09 | | -0.09 | 0.26 | 742 |
| Hemoglobin | |  |  |  |  | 10.94 | 10.86 | -0.09 | | -0.27 | 0.09 | 890 | 10.42 | 10.55 | 0.10 | | -0.11 | 0.30 | 719 |
| Malaria | |  |  |  |  | 0.11 | 0.08 | -0.03 | | -0.07 | 0.01 | 905 | 0.08 | 0.06 | -0.01 | | -0.05 | 0.02 | 729 |

Notes: effect estimates (“T-C”) are from linear regressions with fixed effects for each randomization stratum (25 villages) (Abadie et al 2017).  We do not control for baseline values: for the diarrhea/fever/cough measures, we cannot link individual children across surveys, and no measures were collected at baseline for anthropometry, hemoglobin, and malaria.

**Table S8. Effect of vouchers on social cohesion for households born inside and outside the village**

|  | Coef. | (s.e.) | Obs. |
| --- | --- | --- | --- |
| Households born in the village (six weeks) | 0.19 | (0.18) | 173 |
| Households born in the village (one year) | 0.00 | (0.15) | 139 |
| Households born outside the village (six weeks) | 0.14* | (0.08) | 596 |
| Households born outside the village (one year) | 0.02 | (0.09) | 504 |

Notes: Coefficients are treatment effects are from intention-to-treat analyses with fixed effects for each randomization stratum (25 villages) (Abadie et al 2017). Indices are calculated by rescaling each outcome in each family (e.g. social cohesion) so that higher values imply better outcomes, then standardizing relative to the endline control group, following Kling et al. (2007). If a household was missing an observation for one of the index components, we took the average of the remaining non-missing individual variables for that household. By design, mean values for the control group equal zero and thus are not shown in the table. *p=0.08

**Table S9. Effect of vouchers on psychological well-being at one year, households sharing within dwelling**

|  | Coef. | (s.e.) | Obs. | Control |
| --- | --- | --- | --- | --- |
| *Psychological well-being* | 0.09 | (0.11) | 390 | 0.00 |
| Anxiety/depression | -0.02 | (0.06) | 390 | 1.45 |
| Well-being | 0.00 | (0.09) | 389 | 1.16 |
| Life satisfaction | 0.25* | (0.14) | 384 | 3.07 |

Notes: These estimates compare households that share a dwelling with a treatment household to households that share a dwelling with control household. The coefficients are treatment effects are from intention-to-treat analyses with fixed effects for each randomization stratum (25 villages) (Abadie et al 2017). Indices are calculated by rescaling each outcome in each family so that higher values imply better outcomes, then standardizing relative to the endline control group, following Kling et al. (2007). If a household was missing an observation for one of the index components, we took the average of the remaining non-missing individual variables for that household.

**Deviations from the Pre-Analysis Plan**

This study was preregistered at [Redacted] (6 week) and [Redacted] (one year). Below we discuss deviations from the pre-analysis plan.

- We aimed to collect data from 1,000 households: 100 households in ten sites. Data, however, were collected in seven sites. The reason is that at the moment when the grant period stopped (July 2018), a total of seven EFI voucher interventions had taken place in North Kivu. Between the registration of the pre-analysis plan and the start of the interventions and data collection, we decided to target 140 households per site. We thus targeted a total of 980 households.
- The preregistered social cohesion summary index initially contained additional individual measures to measure also dwelling-related (in addition to the village-related) social cohesion. However, because not all respondents life in multi-household dwellings, we focus on village-related social cohesion only.
- We preregistered analyses of heterogeneous effects across: 1) baseline poverty/vulnerability, 2) migrant/host status, 3) ethnic majority/minority status (relative to village), 4) discordant or concordant ethnicities within the dwelling, 5) assigned voucher amount per capita, 6) occupation of recipient, 7) education of recipient, 8) distance to market and 9) co-residence within dwellings. We do not focus on the heterogeneous effects in this manuscript.
- To adjust for multiple comparisons, we use the free step-down resampling methodology of Westfall and Young (1993) rather than Anderson (2008), because the latter does not account for dependencies across outcomes.

**References**

Abadie, Alberto, Susan Athey, Guido W. Imbens, and Jeffrey Wooldridge. 2017. “When Should You Adjust Standard Errors for Clustering?” National Bureau of Economic Research.

Anderson, Michael L. 2008. “Multiple Inference and Gender Differences in the Effects of Early Intervention: A Reevaluation of the Abecedarian, Perry Preschool, and Early Training Projects.” *Journal of the American Statistical Association* 103 (484): 1481–95.

Kling, Jeffrey R., Jeffrey B. Liebman, and Lawrence F. Katz. 2007. “Experimental Analysis of Neighborhood Effects.” *Econometrica* 75 (1): 83–119. https://doi.org/10.1111/j.1468-0262.2007.00733.x.

Westfall, Peter H., and S. Stanley Young. 1993. *Resampling-Based Multiple Testing: Examples and Methods for p-Value Adjustment*. Vol. 279. John Wiley & Sons.
