# Supplementary material for: Cash-like vouchers improve psychological well-being of vulnerable and displaced persons fleeing armed conflict
Source: PNAS Nexus. 2022 Jun 30;1(3):pgac101. doi: 10.1093/pnasnexus/pgac101 (PMC9896939; doi:10.1093/pnasnexus/pgac101)
Supplement: pgac101_Supplemental_Files [file pgac101_supplemental_files.zip › PNASNEXUS-PNASNEXUS-2022-00251-T-s01.zip!/11_replication/_Readme.docx]

**Replication for “Cash-like vouchers improve psychological well-being of vulnerable and displaced persons fleeing armed conflict”**

June 21, 2022

To replicate all figures, tables and numbers in the text, please follow the following steps:

Have three folders in your replications folder: “Data”, “Temp”, and “Output”

The Data folder should contain:

- The 6-weeks and 1-year survey data. These are publicly available here: <https://dataverse.harvard.edu/dataset.xhtml?persistentId=doi:10.7910/DVN/OPEWXV>
- The folder “igrowup”, which contains data and an ado file created by UNICEF to create anthropometric measures. Downloaded in 2018 from <https://github.com/unicef-drp/igrowup_update>.

Next, run “1_master.do”:

- The only thing to change in this do-file is the location where the replication folder can be found.
- It may also be possible that additional Stata programs need to be installed; which ones are indicated in the file.
- The do-file will call up all the other do-files, pulls up data from the Data folder and outputs in the Temp and Output folders.
- Not all results and information in the text is placed in the Output folder. Some information is provided directly in Stata when running “1_master.do”

**END**
