# Supplementary material for: Cash-like vouchers improve psychological well-being of vulnerable and displaced persons fleeing armed conflict
Source: PNAS Nexus. 2022 Jun 30;1(3):pgac101. doi: 10.1093/pnasnexus/pgac101 (PMC9896939; doi:10.1093/pnasnexus/pgac101)
Supplement: pgac101_Supplemental_Files [file pgac101_supplemental_files.zip › PNASNEXUS-PNASNEXUS-2022-00251-T-s01.zip!/11_replication/Data/igrowup/Readme.pdf]

WHO Child Growth Standards
STATA igrowup package

The package **igrowup_stata** contains the following items:

1. Two macros (***igrowup_standard.ado*** and ***igrowup_restricted.ado***).

2. Nine permanent (read-only) Stata data sets containing the WHO Child Growth Standards: weianthro.dta, lenanthro.dta, wflanthro.dta, wfhanthro.dta, bmianthro.dta, hcanthro.dta, acanthro.dta, tsanthro.dta and ssanthro.dta.

3. The file Readme.pdf

4. An example set, survey.dta.

5. Two example do-files, survey_standard.do and survey_restricted.do.

6. The example output files: mysurvey_z_st.xls, mysurvey_z_rc.xls, mysurvey_z_st.dta, mysurvey_z_rc.dta, mysurvey_prev_st.xls and mysurvey_prev_rc.xls.

Pre-requisites

STATA Version 7.0 Stata/SE (Special Edition of Stata) or higher is required to run two macros (*igrowup_standard.ado* and *igrowup_restriced*). ***Intercooled*** Stata has a limit of 2,047 variables and with that the macros will only produce the z-scores output files (see below the macros' section **Exported files**, point 1). The macros require a STATA data set containing age, sex and the anthropometric measurements. The input variables are specified in the **Parameters** section.

Precautions:

1. Avoid any variable names starting with underscore "_" in the input STATA data set; otherwise they may be replaced by the derived ones created by the macro.

2. Avoid any temporary format names starting with underscore "_"; otherwise they may be replaced by the temporary ones created by the macro.

3. Avoid any STATA global macro variable names staring with underscore "_", except those defined by the system.

Contact for reporting bugs/ comments:

Should you encounter any problems with this package, please send an e-mail with a clear description of the identified problem to "**anthro2005@who.int**", specifying in the subject line that it concerns the ***igrowup_Stata package*** , the name of the macro (*igrowup_standard* or *igrowup_restricted*) and kindly indicate which version of STATA you are using. Thank you.

Recommended setup and run

Step 1. Create a sub-directory, for example "D:\WHO igrowup STATA", where you wish to save the package (**igrowup_stata.zip**). This directory should be reserved only for the references tables (*anthro.dta) and the macros (*igrowup_standard.ado* and *igrowup_restricted*) that are contained in the zip file.

Step 2. Create a sub-directory, for example "D:\WHO igrowup workdata", where the example data (survey.dta and pertaining output files) and your STATA input data can be stored and where all the macro output files will be written to.

Step 3. It is recommended that you start by loading and running the example code below (also found in *survey_standard.do* or *survey_restricted.do*) in the STATA do-file editor to see how the data should be prepared and to fill in the macros' parameters according to their requirements.

Note: The macros run on Stata/SE: The Special Edition version of Stata (type "help SpecialEdition"). For users of Intercooled Stata, the macros will only produce the z scores output files and the user gets the message

Please wait, programme is calculating prevalences............
....
no room to add more variables

References

WHO Multicentre Growth Reference Study Group (2006). WHO Child Growth Standards: Length/height-for-age, weight-for-age, weight-for-length, weight-for-height and body mass index-for-age: Methods and development. Geneva: World Health Organization; pp 312. (available on the web site: http://www.who.int/childgrowth/publications/en/)

WHO Multicentre Growth Reference Study Group (2007). WHO Child Growth Standards: Head circumference-for-age, arm circumference-for-age, triceps skinfold-for-age and subscapular skinfold-for-age: Methods and development. Geneva: World Health Organization; (*forthcoming*)

The macro (igrowup_standard.ado)

Description

1. The macro (***igrowup_standard.ado***) calculates z-scores for the ***eight*** anthropometric indicators, weight-for-age, length/height-for-age, weight-for-length/height, body mass index (BMI)-for-age, head circumference-for-age, arm circumference-for-age, triceps skinfold-for-age and subscapular skinfold-for-age based on the WHO Child Growth Standards. In this macro, all available (non-missing and non-flagged) z-score values are used for each indicator-specific prevalence estimation (standard analysis).

2. The macro produces sex- and age-specific estimates for the prevalence of under/over nutrition and summary statistics (mean and SD) of the z-scores for each indicator.

3. The macro runs with *nine* permanent (read-only) Stata data sets containing the WHO Child Growth Standards: weianthro.dta, lenanthro.dta, wflanthro.dta, wfhanthro.dta, bmianthro.dta, hcanthro.dta, acanthro.dta, tsanthro.dta and ssanthro.dta.

4. Extreme (i.e. biologically implausible) z-scores for each indicator are flagged according to the following system:

Weight-for-age z-score (zwei)	zwei < -6 or zwei > 5
Length/height-for-age z-score (zlen)	zlen < -6 or zlen > 6
Weight-for-length/height z-score (zwfl)	zwfl < -5 or zwfl > 5
BMI-for-age z-score (zbmi)	zbmi < -5 or zbmi > 5
Head circumference-for-age z-score (zhc)	zhc < -5 or zhc > 5
Arm circumference-for-age z-score (zac)	zac < -5 or zac > 5
Triceps skinfold-for-age z-score (zts)	zts < -5 or zts > 5
Subscapular skinfold-for-age z-score (zss)	zss < -5 or zss > 5

Parameters

The macro requires 15 parameters that **must** be specified without any quotation marks.

- ***reflib***: to specify the package directory where the nine STATA data sets containing the WHO Child Growth Standards are stored.

- ***datalib***: to specify the working directory where the input STATA data set containing anthropometric measurements is stored.

- ***datalab***: to specify the name that will prefix the output files (datalab_z_st, datalab_prev_st).

- ***sex:*** to specify the name of a variable containing sex information. If it is a numeric variable, its values must be, 1 for males and 2 for females. And if it is a character variable, it must be, "m" or "M" for males and "f" or "F" for females. Users must code any missing values as "." (for a numeric variable) or " " (for a character variable), in which case no z-scores will be calculated.

- ***age***: to specify the name of a numeric variable containing age information. Age can be in either days or months. An exact age is expected here and should not be rounded if age is in months. Users must code any missing values as "**.**", in which case the age-related z-scores are not calculated.

- ***ageunit***: to specify the age unit of the age variable. It must be specified as either "days" or "months" (they are *case sensitive*). To convert age in months to days, the macro multiplies by 30.4375 and rounds the result to integer for use with the reference tables.

- ***weight***: to specify the name of a numeric variable containing body weight information, which must be in kilograms. Users must code any missing values as "**.**", in which case weight-related z-scores are not calculated.

- ***lenhei***: to specify the name of a numeric variable containing length (recumbent) or height (standing) information, which must be in centimeters. Users must code any missing values as "**.**", in which case length- or height-related z-scores are not calculated. For children aged below 24 months (< 731 days) and measured standing, the macro converts the height to recumbent length by adding 0.7 cm; and for children aged 24 months or above who are measured in recumbent position, the macro converts the length to standing height by subtracting 0.7 cm. In other words, all the z-scores for children below 24 months are length-based, and height-based otherwise. The exported variable ***_clenhei*** is the converted length/ height according to age.

- ***measure***: to specify the name of a character variable indicating whether recumbent length or standing height was measured. The values of this variable must be "l" or "L" for recumbent length, and "h" or "H" for standing height. Users must code any missing values as " ", and the macro imputes any missing values according to the following algorithm:

    a. If age is given, then it is recumbent length if the child's age is below 24 months (< 731 days), and standing height if the child's age is 24 months or above.

    b. If age is missing, then it is recumbent length if ***lenhei*** < 87 cm and standing height if ***lenhei*** ≥ 87 cm.

- ***headc***: to specify the name of a numeric variable containing the head circumference information, which must be in centimeters. Users must code any missing values as **"."**, in which case the head circumference-for-age z-scores are not calculated.

- ***armc***: to specify the name of a numeric variable containing the mid-upper arm circumference information, which must be in centimeters. Users must code any missing values as **"."**, in which case the arm circumference-for-age z-scores are not calculated.

- ***triskin***: to specify the name of a numeric variable containing the triceps skinfold information, which must be in millimeters. Users must code any missing values as **"."**, in which case the triceps skinfold-for-age z-scores are not calculated.

- ***subskin***: to specify the name of a numeric variable containing the subscapular skinfold information, which must be in millimeters. Users must code any missing values as **"."**, in which case the subscapular skinfold-for-age z-scores are not calculated.

- ***oedema***: to specify the name of the character variable containing oedema information. The values of this variable must be "n" or "N" for non-oedema, and "y" or "Y" for oedema. Users must code any missing values as " ", and the macro assumes that they are non-oedema. For oedema cases, weight-related z-scores (_zwei, _zwfl and _zbmi) are not calculated, but they are treated as being < -3 SD in their prevalence estimations.

- ***sw***: to specify the name of a numeric variable containing the sampling weights. If "**sw=1**" for all records, the un-weighted analysis is performed. If otherwise specified, negative values in the sampling weights are not allowed and in this case no prevalence tables will be produced.

Exported files

1. The macro creates, in the working directory, a STATA data set. The name of the data set is ***datalab***_z_st.dta (see the preceding **Parameters** section). This data set retains all the records and variables from the input STATA data set and adds on the following 19 variables derived by the macro:

Variable name	Variable label
_agedays	calculated age in days for deriving z score
_clenhei	converted length/height (cm) for deriving z score
_cbmi	calculated bmi=weight / squared(_clenhei)
_zwei	Weight-for-age z-score
_zlen	Length/height-for-age z-score

_zwfl	Weight-for-length/height z-score
_zbmi	BMI-for-age z-score
_zhc	Head circumference-for-age z-score
_zac	Arm circumference-for-age z-score
_zts	Tricpes skinfold-for-age z-score
_zss	Subscapular skinfold-for-age z-score
_fwei	Flag for _zwei < -6 or _zwe i> 5
_flen	Flag for _zlen < -6 or _zlen > 6
_fwfl	Flag for _zwfl < -5 or _zwf l> 5
_fbmi	Flag for _zbmi < -5 or _zbmi > 5
_fhc	Flag for _zhc < -5 or _zhc > 5
_fac	Flag for _zac < -5 or _zac > 5
_fts	Flag for _zts < -5 or _zts > 5
_fss	Flag for _zss < -5 or _zss > 5

2. The macro creates, in the working directory, a data set in XLS format that has the same data structure as the one in STATA format. The name of the data set is ***datalab***_z_st.xls.

3. The macro creates, in the working directory, an MS Excel file that contains the point estimates and 95% confidence intervals for the indicator prevalences and z-scores summary statistics. Only children aged below 61 months are included in the analysis. The age groups are: Total (0-60), 0-5, 6-11, 12-23, 24-35, 36-47 and 48-60 completed months. The youngest age group is 3-5 months for the arm circumference, triceps and subscapular skinfolds related indicators. Where age is missing, only weight-for-length/height z-scores can be derived and these cases are included in the Total (0-60) age group. The name of this file is *datalab_prev_st.xls*.

```
          /*  Example: survey_standard.do using survey.dta */
clear
set more 1

/*  Higher memory might be necessary for larger datasets */
set memory 20m
set maxvar 10000


/* Indicate to the Stata compiler where the igrowup_standard.ado file is
stored*/
adopath + "D:\WHO igrowup STATA/"

/* Load the data file */
use "D:\WHO igrowup workdata\survey.dta", clear

/* generate the first three parameters reflib, datalib & datalab   */
gen str60 reflib="D:\WHO igrowup STATA"
lab var reflib "Directory of reference tables"
```

```
gen str60 datalib="D:\WHO igrowup workdata"
lab var datalib "Directory for datafiles"

gen str30 datalab="mysurvey"
lab var datalab "Working file"

/*  check the variable for "sex"    1 = male, 2=female */
desc gender
tab gender

/*  check the variable for "age"    */
desc agemons
summ agemons

/*  define your ageunit  */
gen str6 ageunit="months"              /* or gen ageunit="days" */
lab var ageunit "=days or =months"

/*  check the variable for body "weight" which must be in kilograms*/
/*  NOTE: if not available, please create as [gen weight=.]*/
desc weight
summ weight

/*  check the variable for "height" which must be in centimeters*/
/*  NOTE: if not available, please create as [gen height=.]*/
desc height
summ height

/*  check the variable for "measure"*/
/*  NOTE: if not available, please create as [gen str1 measure=" "]*/
desc measure
tab measure

/*  check the variable for "headc" which must be in centimeters*/
/*  NOTE: if not available, please create as [gen headc=.]*/
desc head
summ head

/*  check the variable for "armc" which must be in in centimeters*/
/*  NOTE: if not available, please create as [gen armc=.]*/
desc muac
summ muac

/*  check the variable for "triskin" which must be in millimeters*/
/*  NOTE: if not available, please create as [gen triskin=.]*/
desc tri
summ tri

/*  check the variable for "subskin" which must be in millimeters*/
/*  NOTE: if not available, please create as [gen subskin=.]*/
desc sub
summ sub

/*  check the variable for "oedema"*/
/*  NOTE: if not available, please create as [gen str1 oedema="n"]*/
desc oedema
tab oedema
```

```
/*  check the variable for "sw" for the sampling weight*/
/*  NOTE: if not available, please create as [gen sw=1]*/
desc sw
summ sw

/*  Fill in the macro parameters to run the command */
igrowup_standard reflib datalib datalab gender agemons ageunit weight
height measure head muac tri sub oedema sw
```

The macro (igrowup_restricted.ado)

Description

1. The macro (***igrowup_restricted.ado***) calculates z-scores for the ***four*** anthropometric indicators, weight-for-age, length/height-for-age, weight-for-length/height and body mass index (BMI)-for-age, based on the WHO Child Growth Standards. In this macro, records with at least one flagged z-score (flagged, true missing or with oedema) are excluded to compute the prevalences (restricted analysis).

2. The macro produces sex- and age-specific estimates for the prevalence of under/over nutrition and summary statistics (mean and SD) of the z-scores for each indicator.

3. The macro runs with five permanent (read-only) Stata data sets containing the WHO Child Growth Standards: weianthro.dta, lenanthro.dta, wflanthro.dta, wfhanthro.dta and bmianthro.dta.

4. Extreme (i.e. biologically implausible) z-scores for each indicator are flagged according to the following system:

Weight-for-age z-score (zwei)	zwei < -6 or zwei > 5
Length/height-for-age z-score (zlen)	zlen < -6 or zlen > 6
Weight-for-length/height z-score (zwfl)	zwfl < -5 or zwfl > 5
BMI-for-age z-score (zbmi)	zbmi < -5 or zbmi > 5

Parameters

The macro requires 11 parameters that **must** be specified without any quotation marks.

- ***reflib***: to specify the package directory where the five STATA data sets containing the WHO Child Growth Standards are stored.

- ***datalib***: to specify the working directory where the input STATA data set containing anthropometric measurements is stored.

- ***datalab***: to specify the name that will prefix the output files (*datalab*_z_rc and *datalab*_prev_rc).

- ***sex***: to specify the name of a variable containing sex information. If it is a numeric variable, its values must be, 1 for males and 2 for females. And if it is a character variable, it must be, "m" or "M" for males and "f" or "F" for females. Users must code any missing values as "**.**" (for a numeric variable) or " " (for a character variable), in which case no z-scores will be calculated.

- ***age***: to specify the name of a numeric variable containing age information. Age can be in either days or months. An exact age is expected here and should not be rounded if age is in months. Users must code any missing values as "**.**", in which case the age-related z-scores are not calculated.

- ***ageunit***: to specify the age unit of the age variable. It must be specified as either "days" or "months" (they are *case sensitive*). To convert age in months to days, the macro multiplies by 30.4375 and rounds the result to integer for use with the reference tables.

- ***weight***: to specify the name of a numeric variable containing body weight information, which must be in kilograms. Users must code any missing values as "**.**", in which case weight-related z-scores are not calculated.

- ***lenhei***: to specify the name of a numeric variable containing length (recumbent) or height (standing) information, which must be in centimeters. Users must code any missing values as "**.**", in which case length- or height-related z-scores are not calculated. For children aged below 24 months (< 731 days) and measured standing, the macro converts the height to recumbent length by adding 0.7 cm; and for children aged 24 months or above who are measured in recumbent position, the macro converts the length to standing height by subtracting 0.7 cm. In other words, all the z-scores for children below 24 months are length-based, and height-based otherwise. The exported variable ***_clenhei*** is the converted length/ height according to age.

- ***measure***: to specify the name of a character variable indicating whether recumbent length or standing height was measured. The values of this variable must be "l" or "L" for recumbent length, and "h" or "H" for standing height. Users must code any missing values as " ", and the macro imputes any missing values according to the following algorithm:

    a. If age is given, then it is recumbent length if the child's age is below 24 months (< 731 days), and standing height if the child's age is 24 months or above.

    b. If age is missing, then it is recumbent length if ***lenhei*** < 87 cm and standing height if ***lenhei*** ≥ 87 cm.

- ***oedema***: to specify the name of the character variable containing oedema information. The values of this variable must be "n" or "N" for non-oedema, and "y" or "Y" for

oedema. Users must code any missing values as " ", and the macro assumes that they are non-oedema. For oedema cases, weight-related z-scores (_zwei, _zwfl and _zbmi) are not calculated, but they are treated as being < -3 SD in their prevalence estimations.

- ***sw***: to specify the name of a numeric variable containing the sampling weights. If "**sw=1**" for all records, the un-weighted analysis is performed. If otherwise specified, negative values in the sampling weights are not allowed and in this case no prevalence tables will be produced.

Exported files

1. The macro creates, in the working directory, a STATA data set. The name of the data set is ***datalab***_z_rc.dta (see the preceding **Parameters** section). This data set retains all the records and variables from the input STATA data set and adds on the following 11 variables derived by the macro:

Variable name	Variable label
_agedays	calculated age in days for deriving z score
_clenhei	converted length/height (cm) for deriving z score
_cbmi	calculated bmi=weight / squared(_clenhei)
_zwei	Weight-for-age z-score
_zlen	Length/height-for-age z-score
_zwfl	Weight-for-length/height z-score
_zbmi	BMI-for-age z-score
_fwei	Flag for _zwei < -6 or _zwe i> 5
_flen	Flag for _zlen < -6 or _zlen > 6
_fwfl	Flag for _zwfl < -5 or _zwf l> 5
_fbmi	Flag for _zbmi < -5 or _zbmi > 5

2. The macro creates, in the working directory, a data set in XLS format that has the same data structure as the one in STATA format. The name of the data set is ***datalab***_z_rc.xls.

3. The macro creates, in the working directory, a MS Excel file that contains the point estimates and 95% confidence intervals for the indicator prevalences and z-scores summary statistics. Only children aged below 61 months are included in the analysis. The age groups are: Total (0-60), 0-5, 6-11, 12-23, 24-35, 35-47 and 48-60 completed months. Where age is missing, only weight-for-length/ height z-scores can be derived and these cases are included in the Total (0-60) age group. The name of this file is *datalab_prev_rc.xls.*

```
/*  Example: survey_restricted.do using survey.dta */

clear
set more 1

/*  Higher memory might be necessary for larger datasets */
set memory 20m
set maxvar 10000

/* Indicate to the Stata compiler where the igrowup_restricted.ado file
is stored*/
adopath + "D:\WHO igrowup STATA/"

/* Load the data file */
use "D:\WHO igrowup workdata\survey.dta", clear

/* generate the first three parameters reflib, datalib & datalab   */
gen str60 reflib="D:\WHO igrowup STATA"
lab var reflib "Directory of reference tables"

gen str60 datalib="D:\WHO igrowup workdata"
lab var datalib "Directory for datafiles"

gen str30 datalab="mysurvey"
lab var datalab "Working file"

/*  check the variable for "sex"    1 = male, 2=female */
desc gender
tab gender

/*  check the variable for "age"    */
desc agemons
summ agemons

/*  define your ageunit  */
gen str6 ageunit="months"               /* or gen ageunit="days" */
lab var ageunit "=days or =months"


/*  check the variable for body "weight" which must be in kilograms*/
/*  NOTE: if not available, please create as [gen weight=.]*/
desc weight
summ weight

/*  check the variable for "height" which must be in centimeters   */
/*  NOTE: if not available, please create as [gen height=.]*/

desc height
summ height


/*  check the variable for "measure"*/
/*  NOTE: if not available, please create as [gen str1 measure=" "]*/
desc measure
tab measure
```

```
/*  check the variable for "oedema"*/
/*  NOTE: if not available, please create as [gen str1 oedema="n"]*/
desc oedema
tab oedema

/*  check the variable for "sw" for the sampling weight*/
/*  NOTE: if not available, please create as [gen sw=1]*/
desc sw
summ sw


/*  Fill in the macro parameters to run the command */
igrowup_restricted reflib datalib datalab gender agemons ageunit weight
height measure oedema sw
```
